# An introduced plant affects aquatic-derived carbon in the diets of riparian birds

**DOI:** 10.1371/journal.pone.0207389

**Published:** 2018-11-27

**Authors:** Hannah L. Riedl, Lani Stinson, Liba Pejchar, William H. Clements

**Affiliations:** Department of Fish, Wildlife and Conservation Biology, Colorado State University, Fort Collins, CO, United States of Ameirca; Universidade Regional Integrada do Alto Uruguai e das Missoes, BRAZIL

## Abstract

Non-native plants can impact riparian ecosystem function through diverse terrestrial and aquatic pathways, with cascading effects on food webs. Invasion-mediated vegetation changes can depress terrestrial arthropod communities and alter arthropod flux across the aquatic-terrestrial interface. We investigated the effects of a non-native woody plant, *Robinia neomexicana*, on insect contributions to riparian songbird diets. This plant was introduced over 100 years ago to the Clear Creek drainage in northwestern Colorado (USA) from its native range, which extends into southern Colorado. We used stable isotope analysis of insects and avian feces to 1) assess whether the relative contributions of aquatic- and terrestrial-derived arthropod prey differed between reference sites and sites invaded by *R*. *neomexicana*, and 2) quantify the amount of aquatic- and terrestrial-derived resources consumed by an insectivorous songbird assemblage. Two species of insectivorous songbirds consumed more aquatic insects in invaded sites compared to reference sites. This change in terrestrial- and aquatic-derived prey in bird diets in response to a near-range plant invasion suggests that the introduction of novel species from more distant native ranges could produce similar or stronger effects. Overall, the songbird community consumed approximately 34% aquatic resources, which highlights the importance of these subsidies to riparian consumers. Our investigation of insect subsidies demonstrates how introduced species can indirectly affect food webs and provides insight into the plasticity of riparian consumer responses.

## Introduction

Invasive species impact ecosystem structure and function [1] and lead to biotic homogenization of communities [2]. Characteristics related to the life history, physiology and chemistry of invasive plants can drive fundamental shifts in primary production, nutrient cycling, water usage, and decomposition [1, 3–5]. Recent syntheses have attempted to identify patterns in the mechanisms and consequences of invasion across diverse ecosystems, taxa and levels of ecological complexity [6–8]. Few consistent trends emerged from these assessments. Instead, the effects of introduced plants appear to be highly context-specific, varying in direction and magnitude across ecosystems, taxa, and functional traits [6–8]. In particular, the extent to which near-range introductions alter ecosystem processes remains unclear [9, 10]. Invasive species studies also tend to focus on a single ecosystem function and fail to address the interacting and potentially reinforcing mechanisms underlying invasion-driven ecosystem change [11]. Resource subsidies, or fluxes of resources between ecosystems, can provide insight into the multiple ways invasive species alter biological communities [12, 13].

Riparian plant and animal communities are particularly susceptible to invasion, which can lead to impacts on ecosystem services and human well-being [14, 15]. New assemblages of taxa, driven by species invasions and climate change, can alter the timing, duration, and magnitude of aquatic or terrestrial insect subsidies [16–18], and these factors can interact to decouple cross-ecosystem processes [19]. Changes in the riparian forest canopy, for example, can affect aquatic insect communities by altering litter input quality and quantity, canopy openness, and algal communities [3, 20]. Furthermore, compared to native vegetation, non-native plants often support decreased terrestrial arthropod abundance, biomass and richness [21, 22]. Changes in the relative availability of aquatic or terrestrial insects have the potential to cascade through food webs, with bottom-up impacts on riparian insectivorous birds [3, 23].

Riparian bird assemblages may be sensitive to invasion-mediated changes in cross-ecosystem subsidies because many insectivorous species consume aquatic prey. In particular, aquatic insects contribute substantially to avian insectivore diets, and some species are entirely dependent on aquatic food resources during certain times of year [24, 25]. Differences in the diet composition (i.e., proportion of aquatic versus terrestrial prey) of birds using riparian habitats dominated by either native or introduced plants may reflect invasion-mediated diet shifts, with potential consequences for the integrity and persistence of diverse riparian bird communities [26]. Yet, despite the susceptibility of riparian areas to invasive species [27], few studies have addressed the potential effects of plant invasion on insect subsidies provided to avian consumers.

This study evaluated how an introduced plant, *Robinia neomexicana* (New Mexico locust), altered the aquatic- and terrestrial-derived diet components of riparian bird diets and quantified the contribution of aquatic-derived carbon to insectivorous songbird diets. *R*. *neomexicana* is native to the southwestern United States and its native range extends into portions of southern Colorado [28, 29]. This species was introduced >100 years ago to an area north of its native range in the Clear Creek drainage of the Piceance Basin of northwestern Colorado, USA, where it has become well-established and dominant in some reaches of the watershed. Functional traits, such as rhizomatous growth and the ability to fix nitrogen, likely make *R*. *neomexicana* a successful pioneer species. Landowners have attempted to remove the plant, with little success in limiting or reducing spread (C. Tysse, Chevron, personal communication).

To determine whether invasion by *R*. *neomexicana* affected riparian consumers via altered resource subsidies, we examined the diets of insectivorous songbirds captured from reference and invaded sites using stable isotope analysis (SIA) of fecal samples. Primary producers in aquatic and terrestrial ecosystems often have distinct *δ*^13^C values because of variation in plant physiology and resource availability, and these tracers exhibit little isotopic fractionation during trophic transfer [30, 31]. Naturally abundant isotopes of carbon (^13^C) can be used to track time-integrated contributions of aquatic- and terrestrial-derived energy through food webs [32]. Isotopes of nitrogen (^15^N) can be used to track differences in food web structure because consumers typically become enriched in ^15^N with increasing trophic position [33, 34]. We predicted that songbird fecal samples in invaded sites would have *δ*^13^C signatures more similar to aquatic-derived prey signatures, indicating decreased reliance on terrestrial insects. Non-native vegetation often supports depauperate terrestrial arthropod communities compared to native plants [21, 22, 35], and songbirds often forage in proportion to prey availability [36, 37]. Therefore, we hypothesized that fecal samples from invaded sites would be less enriched in *δ*^15^N because vegetation invasions often disproportionately reduce higher trophic level arthropods compared to lower trophic level taxa [22, 38].

We also used SIA to quantify the proportion of aquatic-derived prey in songbird diets. We predicted that aquatic insects would contribute to the diets of riparian songbirds, but that reliance on this resource subsidy would vary among species [25, 26, 39]. Specifically, we hypothesized that diets of year-round insectivores would have higher proportions of aquatic-derived prey than more omnivorous species. Our results reveal that a large proportion of songbird diets are derived from aquatic resources, and that plant invasions, even those occurring near their native ranges, can alter the diet of insectivorous songbirds, with potential for cascading effects throughout riparian food webs.

## Methods

### Study area

This study was conducted on private land in the Clear Creek drainage of northwestern Colorado, USA (39.5°N, 108.2°W), located approximately 60 km northeast of Grand Junction. Permission to conduct the study was granted by the private landowners (C. Tysse, Chevron, personal communication). The area has undergone significant oil and gas development, with oil pads and other infrastructure near the riparian zone, including a gravel access road paralleling the main stem of Clear Creek. The landscape is topographically diverse (1500–2700 m elevation) and is characterized by high mesas and steep canyons surrounding the 1^st^ and 2^nd^ order streams of Clear Creek and its tributaries. The riparian corridor within the study area averaged 49 ± 8 m SE in width and was dominated by native trees including *Acer negundo* (boxelder), *Populus angustifolia* (narrowleaf cottonwood), and *Quercus gambelii* (Gambel oak), as well as *R*. *neomexicana* in invaded areas. The shrub layer consisted of *Amelanchier alnifolia* (serviceberry), *Artemisia tridentate* (big sagebrush), *Ericameria* sp. (rabbitbrush), *Symphoricarpos* sp. (snowberry), *Prunus virginiana* (chokecherry), *Ribes* sp. (currant), and *Rosa woodsia* (wood’s rose).

### Sampling design

**Fig 1:**
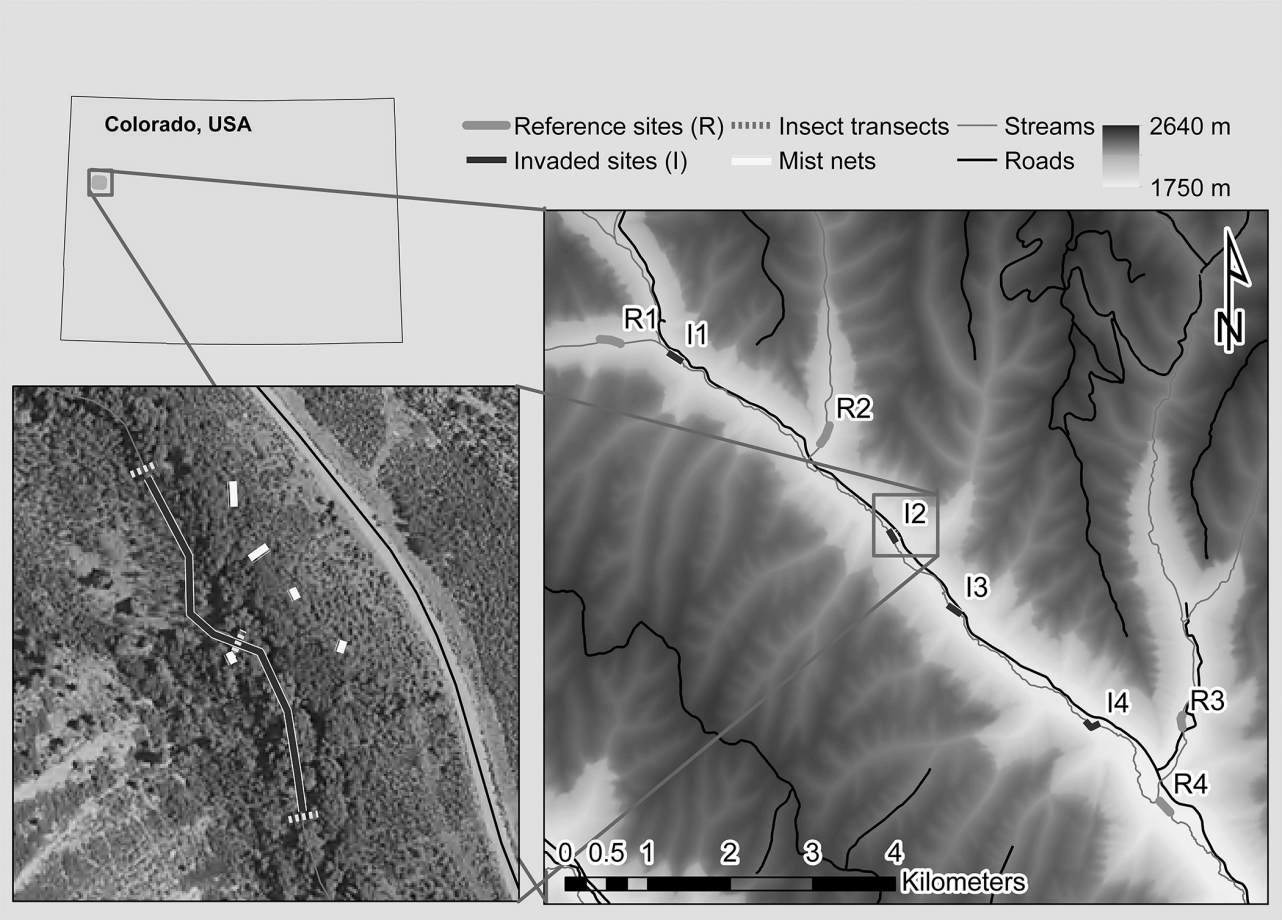
Study area and sampling reach map. Insect and bird sampling sites along uninvaded (reference) and invaded stream reaches in the Clear Creek drainage of northwestern Colorado, USA. The lower left inset illustrates mist net locations where fecal samples were collected, and insect transects where insect samples were collected for an individual site.

After an initial reconnaissance mapping of stream reaches, we established eight 180 m sampling sites within reference and invaded reaches, spaced at least 300 m apart and paired by elevation (Fig 1). Streams within study reaches were relatively narrow (2.3 ± 1.1 m SE) and of moderate gradient (2.2 ± 1.7% SE, measured from four repeated GPS coordinate measurements), with fine silts and small gravel typical of the oil shale geology of the region.

### Insect sampling

Aquatic and terrestrial insects in reference and invaded sites were sampled by deploying emergence and pan traps on the upstream, middle, and downstream transect of each reach. We placed one floating emergence net (0.2-mm mesh, 0.3 m^2^) over slow-moving water nearest each transect to capture adult aquatic insects emerging from the stream channel [40]. We placed one pan trap (0.4 m^2^) over each transect above the water’s surface to capture adult aquatic insects and terrestrial arthropods falling into the stream. The pan traps were filled 5 cm deep with stream water and approximately 5 mL of biodegradable surfactant was added to reduce surface tension [41]. Both trap types were deployed simultaneously for 48 hours (2015: 21–22 July; 2016: 30–31 July) and collected insects were preserved in 70% ethanol. This timeframe for sampling insects was selected to align with songbird fecal sampling because it was essential to minimize disturbance during the nesting season.

We enumerated and identified all insects collected to family using taxonomic keys [42, 43]. We selected dominant herbivorous terrestrial and aquatic insects based on mean percent of individuals in pan traps or emergence nets pooled across years. Dominant herbivorous terrestrial taxa included Cicadellidae (9.6%), Lepidopteran larvae (5.8%) and Aphididae (0.44%). Emergent aquatic insect richness averaged only two taxa per sample (a maximum of six taxa in one sample) throughout the study, so we considered all functional feeding groups except shredders as candidates for isotope analysis (i.e., filter feeders, collector-gatherers, and algae grazers). Shredders were excluded because they feed on terrestrial leaf litter inputs, and thus would have *δ*^13^C signatures similar to terrestrial-derived carbon. Dominant aquatic taxa in emergence nets included Chironomidae (57.5%), Simuliidae (7.7%, 2015), Heptageniidae (7.5%, 2016), and Baetidae (6.1%). Heptageniidae were substituted for Simuliidae in 2016 because only 1 individual simuliid occurred in all samples in 2016.

### Songbird fecal sampling

Mist-netting of insectivorous songbirds to collect fecal samples for SIA was undertaken in late summer of each year (2015: 11 July–5 August; 2016: 17 July–6 August), corresponding with the time that insects were sampled. All required state and federal permits associated with mist-netting and fecal sample collection were obtained prior to field sampling. Sampling procedures were reviewed and approved by the Institutional Animal Care and Use Committee (IACUC) at Colorado State University. We sampled late in the songbird breeding season to minimize disturbance to nesting pairs and allow capture of adults and fledged juveniles prior to migration. Within each of the 8 sampling sites, we set up 4–7 mist nets (38-mm mesh, 6–12 m), with the goal of maintaining approximate equal sampling effort between reference and invaded sites. Nets were placed along habitat edges and bisecting the riparian corridor. We opened nets for 2–4 days per site during the morning hours when weather conditions allowed safe capture and removal of songbirds; nets were closed during times of rain or high wind. Each captured bird was removed from the mist net and placed in a cloth bag for several minutes to allow time for defecation into the bag. Fecal samples were collected from the bags and stored in 70% alcohol in individually labeled vials for later processing. Songbird feces contain insects ingested within a few hours before capture, making them ideal for examining diet changes over small spatial and short time scales [44]. Using feces for dietary analysis is also a less invasive alternative to stomach lavage or tissue sampling [44–46].

### Stable isotope processing

Fecal and insect samples were dried at 60°C for 48 hours, homogenized, and weighed to a precision of 0.001 mg into 4 x 6 mm cylindrical tin capsules. Stable isotopes were measured at the Natural Resource Ecology Laboratory (Colorado State University, Fort Collins, Colorado, USA) using a Carlo Erba NA 1500 (Milan, Italy) coupled with a VG Isochrom continuous flow isotope ratio mass spectrometer (Isoprime Inc., Manchester, United Kingdom) to simultaneously determine nitrogen and carbon isotope composition. Ratios of the heavy isotope to its common lighter counterpart (i.e., ^13^C/^12^C and ^15^N/^14^N) were expressed in standard *δ*-notation relative to international standards (Vienna Peedee Belemnite and atmospheric nitrogen, respectively) in parts per mil (‰). For instance, *δ*^13^C_sample_ = [(^13^C_sample_/^12^C_sample_)/(^13^C_standard_/^12^C_standard_)-1] x 1000, and likewise for *δ*^15^N [47]. Analytical precision from multiple in-house runs was 0.2 ‰ for *δ*^13^C and 0.3 ‰ for *δ*^15^N.

### Statistical analyses

#### Invasion-mediated diet shifts

We conducted species-specific multivariate analyses to examine invasion-mediated diet shifts, which we defined as differences in songbird diet isotope signatures between reference and invaded sites. We analyzed fecal samples from seven songbird species, including five year-round insectivores and two omnivorous species whose diets are dominated by insects during the breeding season (S1 Table) [48]. We considered the two species of flycatchers, Cordilleran flycatcher (*Empidonax occidentalis)* and dusky flycatcher (*Empidonax oberholseri)*, as a single unit (flycatcher). Analyses were conducted separately for each year to account for known annual variation in arthropod communities in this watershed [35].

We tested for songbird diet shifts between reference and invaded sites using one-way MANOVAs with *δ*^13^C and *δ*^15^N as dependent variables and site type as the independent variable. All MANOVAs were conducted with SAS PROC GLM. Isotope data were normally distributed, and Satterthwaite degrees of freedom were used to correct for unequal variance where necessary. F-values from MANOVAs are reported from Wilks’ Lambda criteria. ANOVAs were considered to determine whether differences were driven by *δ*^13^C (diet source) or *δ*^15^N (diet position).

#### Aquatic-derived carbon in songbird diets

To identify the relative contributions of aquatic- and terrestrial-derived prey in the diets of songbirds, we used *δ*^13^C of fecal and insect samples in a single-isotope mixing formula [47]. SIA of insect samples provides context for *δ*^13^C shifts in songbird diets and provides terms in the mixing formula used to calculate the proportions of aquatic and terrestrial diet components for each fecal sample. First, we tested for differences between site types in *δ*^13^C of insect samples to determine appropriate grouping for calculation of diet sources. *δ*^13^C of insect samples were evaluated using two-way analysis of variance (ANOVA) by year with *δ*^13^C as the dependent variable and site type (2 levels, fixed effect), species (6 levels, fixed effect), and the interaction as independent factors in the model. ANOVA was conducted using SAS v9.3 (SAS Institute, Cary, North Carolina, USA) PROC MIXED. For both 2015 and 2016, *δ*^13^C isotopic signatures of insect samples were not statistically different between site types (p > 0.15), although there was significant species-specific variation (p < 0.02, S1 Fig). Therefore, *δ*^13^C signatures of aquatic and terrestrial insects were determined as an average of the three dominant aquatic and terrestrial taxa, respectively, across all sites for each year.

Next, we used a mixing formula to identify the relative proportions of aquatic- and terrestrial-derived insects in songbird diets [47]:

$$p_{1}=\left(\delta_{sample}-\delta_{source,2}\right)/\left(\delta_{source,1}-\delta_{source,2}\right)\;and$$

$$p_{2}=1‑p_{1}$$

where *δ*_sample_ is the *δ*^13^C value of each fecal sample, *p*_i_ is the proportion of aquatic or terrestrial diet sources, and *δ*_source,i_ is the average *δ*^13^C for each diet source [47]. In instances of a “mixing muddle” [47], where the fecal sample occurred outside the range characterized by aquatic and terrestrial insect isotope signatures, we classified the sample as composed entirely of the diet source the sample most closely resembled.

Because we selected dominant insects feeding primarily on aquatic- and terrestrial-derived primary producers, our source samples did not reflect signatures of higher trophic level arthropods (e.g., predaceous spiders, parasitic wasps). Additionally, inclusion of filter-feeding aquatic insects, such as Simuliidae, as candidates for SIA could bias the aquatic-derived signature towards *δ*^15^N enrichment since this feeding guild incidentally ingests animal parts. Thus, we did not use *δ*^15^N of insect samples to quantify songbird diet components (S1 Fig).

## Results

### Invasion-mediated diet shifts

**Fig 2:**
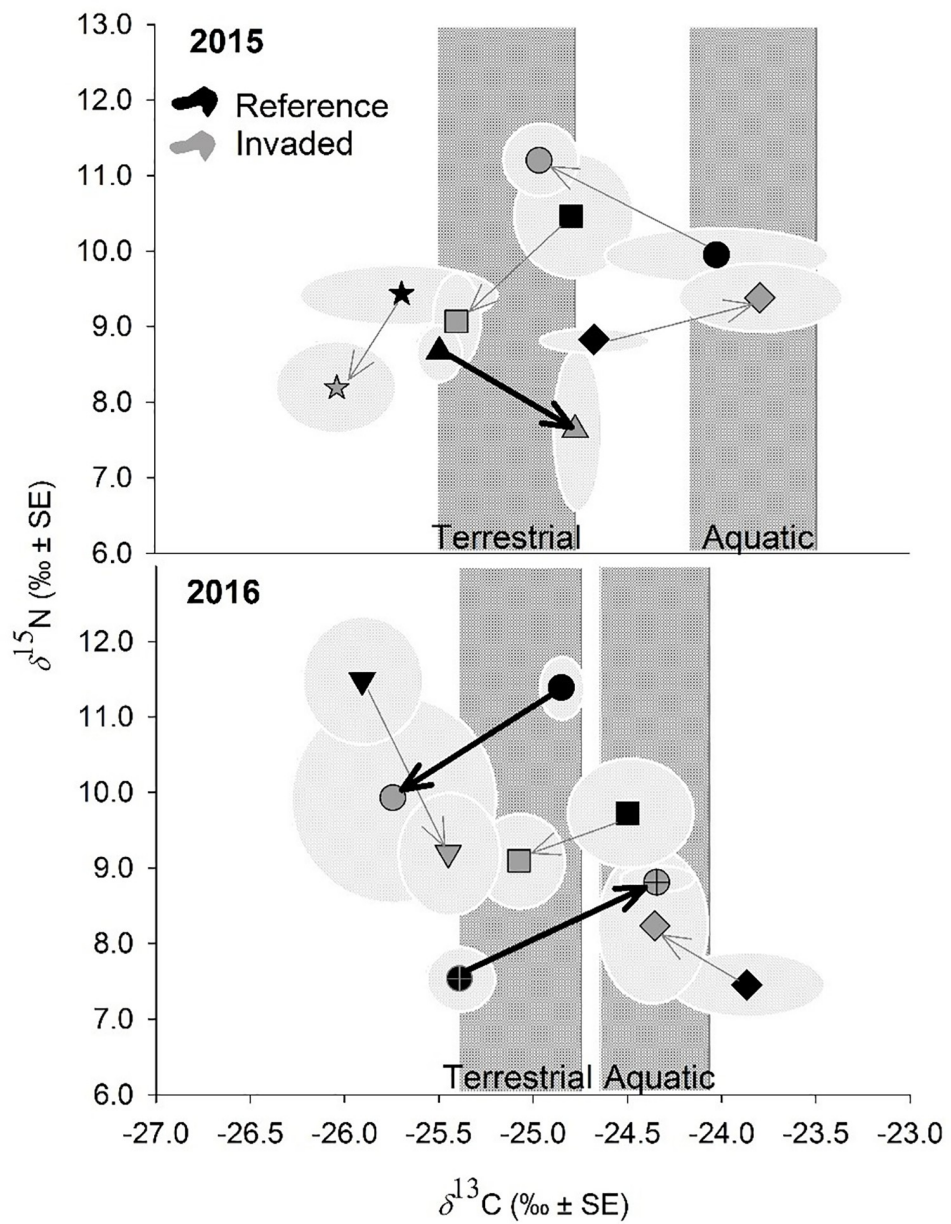
Carbon isotopes suggest some songbird species consume more aquatic insects in riparian areas invaded by *R*. *neomexicana*. Biplots show *δ*^13^C and *δ*^15^N signatures of songbird fecal samples, illustrating significant invasion-mediated diet shifts in three species (bold arrows; Table 1). Arrows connect reference to invaded sites for each species sampled in the Clear Creek drainage of northwestern Colorado, USA. Dark gray shading shows the standard error of the *δ*^13^C signatures of terrestrial and aquatic insect samples. Light gray ellipses represent the standard error encompassed by *δ*^13^C and *δ*^15^N signatures of songbird fecal samples. Flycatchers (○), yellow warblers (□), and green-tailed towhees (◊) were evaluated in both years. MacGillivray’s warblers (✩) and Virginia’s warblers (△) were only evaluated in 2015, and warbling vireos (⨁) and black-capped chickadees (▽) were only evaluated in 2016.

We collected and analyzed isotopic signatures of 133 fecal samples from 7 species of songbirds. All 7 species were sampled at reference and invaded sites during the study, and 53 of the fecal samples were obtained in reference sites (Fig 2; S1 Table). All significant diet shifts were driven by diet source (*δ*^13^C). This finding reflects differences in the relative reliance on aquatic- and terrestrial-derived prey resources in reference and invaded sites, although other diet shift patterns varied among songbird species and between years. No differences in *δ*^15^N signatures were detected for any species evaluated, indicating diet shifts were not driven by trophic position or altered populations of predaceous or parasitic arthropods in invaded sites. Of the seven species examined, Virginia’s warblers (*Leiothlypis virginiae*) showed a significant invasion-mediated diet shift towards aquatic-derived carbon in 2015 (p = 0.021), and warbling vireos *(Vireo gilvus)* showed a significant diet shift towards aquatic-derived carbon in 2016 (p = 0.023, Fig 2; Table 1). In contrast, flycatchers showed a significant invasion-mediated diet shift that trended towards more terrestrially-derived carbon and less *δ*^15^N enrichment in 2016 (p = 0.002). However, it is unclear if this shift was driven by *δ*^13^C or *δ*^15^N because separate univariate analyses revealed no statistical differences in either signature individually (Table 1). Multivariate approaches test for differences in the combined effects of dependent variables and, therefore, can detect differences too slight for univariate analyses. Yellow warblers *(Setophaga petechia)* and green-tailed towhees (*Pipilo chlorurus*) showed no diet shifts consistently across years, and MacGillivray’s warblers *(Geothlypis tolmiei)* and black-capped chickadees *(Poecile atricapillus)* showed no diet shifts in the single years they were evaluated (Table 1).

**Table 1:** One-way MANOVAs and ANOVAs by year, testing for differences in songbird diet *δ*^13^C and *δ*^15^N signatures between reference and invaded sites. Only species with multiple samples per site/year combination were evaluated (dashes in place otherwise). Significant differences (p<0.05) are shown in bold. Fig 3 shows directionality of significant diet shifts with bold arrows.

	*δ*^13^C and *δ*^15^N (MANOVA)				*δ*^13^C (ANOVA)				*δ*^15^N (ANOVA)			
	2015		2016		2015		2016		2015		2016	
Songbird Species	F	*p*	F	*p*	F	*p*	F	*p*	F	*p*	F	*p*
Flycatcher	1.28	*0*.*32*	**28.2**	***0*.*002***	0.81	*0*.*38*	2.52	*0*.*16*	2.13	*0*.*17*	1.08	*0*.*34*
Warbling Vireo	-		**19.4**	***0*.*019***	-		**13**	***0*.*023***	-		4.37	*0*.*10*
Black-capped Chickadee	-		1.43	*0*.*29*	-		0.8	*0*.*38*	-		2.9	*0*.*12*
MacGillivray's Warbler	0.64	*0*.*55*	-		0.31	*0*.*59*	-		1.41	*0*.*27*	-	
Virginia's Warbler	4.41	*0*.*067*	-		**8.79**	***0*.*021***	-		0.23	*0*.*64*	-	
Yellow Warbler	2.72	*0*.*10*	1.64	*0*.*24*	3.89	*0*.*069*	1.7	*0*.*22*	1.85	*0*.*19*	0.42	*0*.*53*
Green-tailed Towhee	0.38	*0*.*70*	0.54	*0*.*60*	0.51	*0*.*49*	0.79	*0*.*40*	0.24	*0*.*63*	0.61	*0*.*45*

### Aquatic-derived carbon in songbird diets

**Fig 3:**
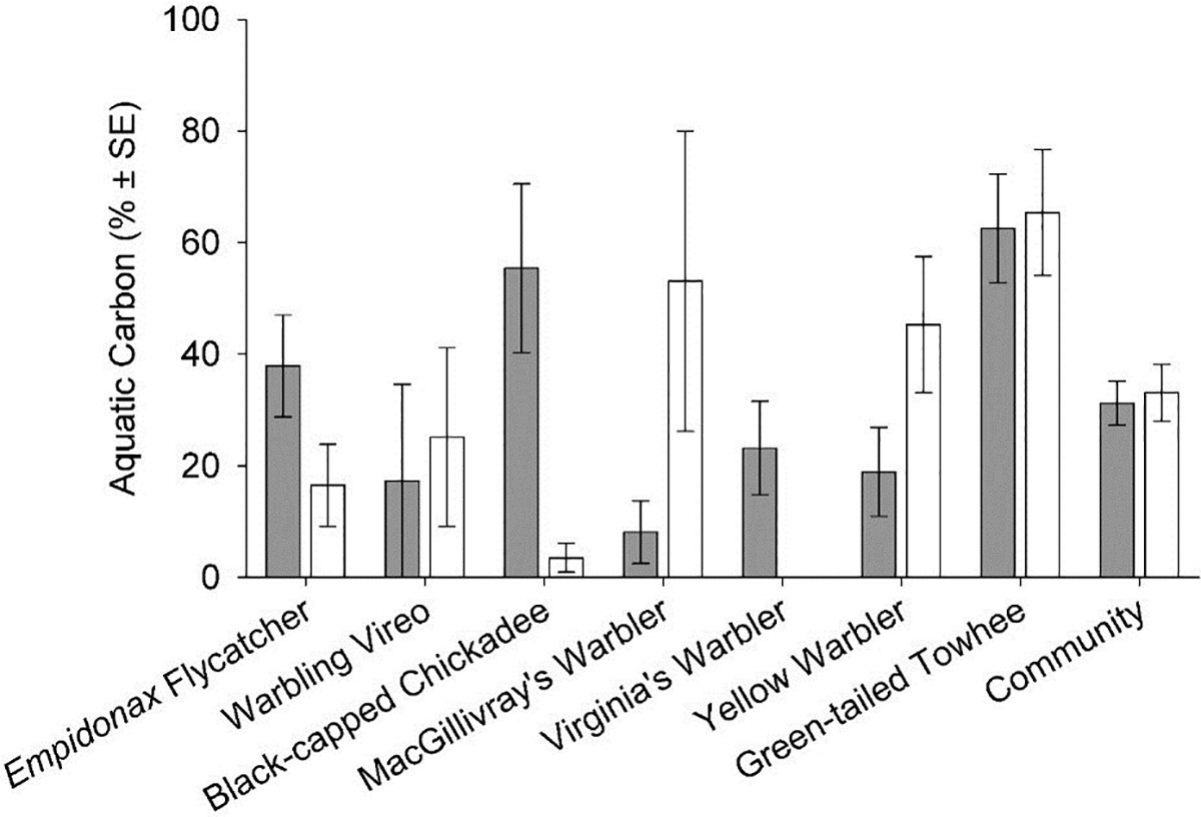
Contributions of aquatic-derived insect carbon to the diets of songbird species. The figure shows mean percent aquatic-derived carbon sampled from songbird species individually and overall (i.e., “community”) for 2015 (grey) and 2016 (white). Results were determined using a single isotope mixing formula and *δ*^13^C signatures of insect and fecal samples, pooled across reference and invaded sites. There were insufficient sample sizes (n<2 per year) to calculate means for Virginia’s warbler in 2016.

Overall, the songbird community consumed 34 ± 3% SE aquatic-derived carbon throughout the study, with no difference between years or site types (Fig 3; S2 Table). The total contribution (across both years) of aquatic-derived carbon to insectivore diets varied among species, ranging from 18% for MacGillivray’s warblers to 64% for green-tailed towhees (S2 Table).

The *δ*^13^C signature of insect samples was not statistically different between reference and invaded sites during 2015 and 2016 (p > 0.15), although there was significant species-specific variation (p < 0.02, S1 Fig). Insects collected from aquatic systems were more enriched in *δ*^13^C than their terrestrial counterparts (i.e., less negative *δ*^13^C isotopic signatures). While the overall contribution of aquatic-derived insects to the diet of the songbird assemblage was similar between years, species-specific values often differed (Fig 3; S2 Table). For example, black-capped chickadees consumed mostly aquatic carbon in 2015, and mostly terrestrial carbon in 2016, and these trends were opposite for MacGillivray’s warblers. During both years, however, flycatchers, warbling vireos, and yellow warblers consumed more terrestrial carbon, and green-tailed towhees consumed more aquatic carbon.

## Discussion

Significant shifts in the diets of two songbirds lends support to our hypothesis that some bird species would rely more on aquatic insects at invaded sites (Fig 2; Table 1). These species, Virginia’s warbler and warbling vireo, are foliage gleaners that may be more impacted by the reductions in terrestrial insects associated with invasion [21, 22, 35, 48]. Although we did not detect significant diet shifts associated with trophic position (i.e., driven by *δ*^15^N) for any bird species evaluated, this is consistent with findings of Riedl et al. [35], which found no significant difference in the abundance of predaceous or parasitic arthropods between sites. The timing of our sampling in the late summer likely influenced our findings [25]. However, it was necessary to avoid capturing individuals earlier in the season during times of peak egg-laying and nest incubation, when the risk of nest abandonment is higher. Future work that includes sampling in spring may find more pronounced invasion-mediated diet shifts for systems invaded by plants with a later leaf-out phenology than native vegetation. In our system, delayed timing of *R*. *neomexicana* leaf-out likely provides less foliage to support arthropod production in late spring [35]. Our study was the first to use SIA to detect songbird diet shifts in response to a riparian plant invasion. However, the scope of our study was temporally and spatially limited. Additional research spanning longer timeframes and conducted in a diversity of riparian systems is warranted to more fully evaluate broad support for our findings.

We demonstrate that the diet of insectivorous songbirds in our riparian study system consisted of approximately 1/3 aquatic insects and 2/3 terrestrial insects during summer months. This finding highlights the importance of maintaining intact riparian systems and productive aquatic resource subsidies for terrestrial consumers. Our estimate of the aquatic insect contribution to songbird diets is somewhat higher than other published estimates. Along the Colorado River in Arizona, USA, aquatic insects accounted for only 9% of the diet of insectivorous songbirds during summer months [39]. In temperate riparian forests in Japan, aquatic prey consumed in summer by a diverse bird assemblage averaged 6% (range: 0–29%, n = 18 species), with flycatchers and warblers consuming the highest percentages of aquatic arthropods [25]. However, community-level estimates are strongly influenced by the species composition and foraging habits of songbirds included in an assemblage. Overall, year-round insectivores in our study system did not consume a higher proportion of aquatic-derived carbon. In contrast, Green-tailed towhees, which are insectivorous only in the breeding season, consumed the most aquatic-derived carbon in both years.

We assumed that insectivorous songbirds consume prey in proportion to what is available, exhibiting prey switching in response to reductions in preferred prey resources [25, 36, 37]. Therefore, alterations in the availability of insects from different sources or trophic levels should translate into shifts in *δ*^13^C or *δ*^15^N, respectively. Because we did not detect differences in insect *δ*^13^C or *δ*^15^N signatures between reference and invaded sites, diet shifts likely did not result from invasion-mediated changes to prey signatures. It is possible, however, that insectivorous birds modified their foraging strategies in response to altered resource subsidies [39]. Rather than switch prey, birds may forage more efficiently for preferred prey resources, and this change in behavior would not be detected by SIA. Furthermore, studies evaluating food web impacts on other consumer taxa do not always reveal changes in diets consistent with alterations in prey subsidies [49, 50]. For example, despite large reductions in terrestrial arthropod biomass in watersheds invaded by European bird cherry (*Prunus padus*), Roon, Wipfli (49] found no difference in the proportion of terrestrial insects in the diets of juvenile coho salmon (*Oncorhynchus kisutch*).

Our study sites contained diverse arthropod prey communities; however, we are confident our selection of dominant insects sufficiently described prey availability. Multiple studies have reported that Chironomidae (or other Diptera), Cicadellidae, and Lepidopteran larvae comprise the majority of prey for insectivorous riparian birds, including upper-canopy gleaners like yellow warblers [37, 39, 51]. However, our inability to measure isotopic signatures of all available prey items limits interpretation of consumer isotope data in relation to prey items. Pan trap sampling may not have captured a representative sample of the prey items available to avian insectivores, such as insects gleaned off vegetation. Thus, our estimates of aquatic- and terrestrial-derived diet proportions should be considered a general index rather than an exact proportion.

### Conclusions

Using isotopic signatures of insects and fecal samples, we found support that diet shifts towards aquatic-derived carbon were associated with plant invasion for two insectivorous bird species. Diet shifts between reference and locust-invaded habitats were inconsistent for other bird species and between years. We estimated that the riparian songbird community consumed 34% aquatic carbon, which highlights the importance of aquatic resource subsidies to terrestrial consumers. These diet shifts occurred in a watershed near the introduced plant’s native range, which suggests that species introduced from more geographically disparate areas could have similar or more pronounced impacts on riparian food webs [9, 10]. An increased focus on resource subsidies will provide a more mechanistic understanding of the consequences of anthropogenic change by examining interacting processes across ecosystems.

## Supporting information

S1 Table Sample sizes. Number of fecal samples collected from songbird species at sites uninvaded (reference) and invaded by R. neomexicana in the Clear Creek drainage of northwestern Colorado, USA. Common names of songbird species are listed in taxonomic order. Cordilleran flycatchers and dusky flycatchers were analyzed together as flycatchers. Black-capped chickadees and green-tailed towhees are omnivores that are insectivorous during the breeding season, while the remaining five species are year-round insectivores [48].
(DOCX)

S2 Table Aquatic carbon diet components. Comparison of aquatic carbon contributions to songbird diets across species and years, based on data pooled across reference and invaded sites. Mean estimates of % aquatic carbon ± SE were calculated using a single isotope mixing formula and *δ*^13^C signatures of insect and fecal samples. The terrestrial-derived diet component is the remaining percentage (1 –aquatic percentage).
(DOCX)

S1 Fig Insect isotopes. Biplots showing *δ*^13^C and *δ*^15^N signatures of aquatic (gray) and terrestrial insect taxa (black) used as an index of songbird diet sources in 2015 (top) and 2016 (bottom). Common names of taxa are displayed near the mean.
(TIF)

S1 Text Raw data used for all analyses. (XLSX)
