# Supplementary material for: An introduced plant affects aquatic-derived carbon in the diets of riparian birds
Source: PLoS One. 2018 Nov 27;13(11):e0207389. doi: 10.1371/journal.pone.0207389 (PMC6258477; doi:10.1371/journal.pone.0207389)
Supplement: S1 Table — Number of fecal samples collected from songbird species at sites uninvaded (reference) and invaded by R. neomexicana in the Clear Creek drainage of northwestern Colorado, USA. Common names of songbird species are listed in taxonomic order. Cordilleran flycatchers and dusky flycatchers were analyzed together as flycatchers. Black-capped chickadees and green-tailed towhees are omnivores that are insectivorous during the breeding season, while the remaining five species are year-round insectivores [48]. (DOCX) [file pone.0207389.s001.docx]

| **Songbird Species** | **Reference** | | | **Invaded** | | |
| --- | --- | --- | --- | --- | --- | --- |
|  | *2015* | *2016* | *Total* | *2015* | *2016* | *Total* |
| Flycatcher | 6 | 4 | 10 | 12 | 4 | 16 |
| Warbling Vireo | 1 | 4 | 5 | 2 | 2 | 4 |
| Black-capped Chickadee | 0 | 8 | 8 | 8 | 4 | 12 |
| MacGillivray's Warbler | 3 | 2 | 5 | 8 | 1 | 9 |
| Virginia's Warbler | 2 | 1 | 3 | 7 | 0 | 7 |
| Yellow Warbler | 6 | 7 | 13 | 10 | 6 | 16 |
| Green-tailed Towhee | 2 | 7 | 9 | 11 | 5 | 16 |
| **All Species Combined** | **20** | **33** | **53** | **58** | **22** | **80** |
