# Supplementary material for: An introduced plant affects aquatic-derived carbon in the diets of riparian birds
Source: PLoS One. 2018 Nov 27;13(11):e0207389. doi: 10.1371/journal.pone.0207389 (PMC6258477; doi:10.1371/journal.pone.0207389)
Supplement: S2 Table — Comparison of aquatic carbon contributions to songbird diets across species and years, based on data pooled across reference and invaded sites. Mean estimates of % aquatic carbon ± SE were calculated using a single isotope mixing formula and δ13C signatures of insect and fecal samples. The terrestrial-derived diet component is the remaining percentage (1 –aquatic percentage). (DOCX) [file pone.0207389.s002.docx]

| **Songbird Species** |  | **2015** | |  | **2016** | |  | **Total** | |
| --- | --- | --- | --- | --- | --- | --- | --- | --- | --- |
|  |  | *n* | *Mean % Aq.* ± SE |  | *n* | *Mean % Aq.* ± SE |  | *n* | *Mean % Aq.* ± SE |
| Flycatcher |  | 18 | 38 ± 9 |  | 8 | 17 ± 7 |  | 26 | 31 ± 7 |
| Warbling Vireo |  | 3 | 17 ± 17 |  | 6 | 25 ± 16 |  | 9 | 22 ± 12 |
| Black-capped Chickadee |  | 8 | 55 ± 15 |  | 12 | 3 ± 3 |  | 20 | 24 ± 8 |
| MacGillivray's Warbler |  | 11 | 8 ± 6 |  | 3 | 53 ± 27 |  | 14 | 18 ± 8 |
| Virginia's Warbler |  | 9 | 23 ± 8 |  | 1 | - |  | 10 | 24 ± 8 |
| Yellow Warbler |  | 16 | 19 ± 8 |  | 13 | 45 ± 12 |  | 29 | 31 ± 7 |
| Green-tailed Towhee |  | 13 | 62 ± 10 |  | 12 | 65 ± 11 |  | 25 | 64 ± 7 |
| **Community** |  | **78** | **33 ± 4** |  | **55** | **34 ± 5** |  | **133** | **34 ± 3** |
