# Supplementary figures and images for: An introduced plant affects aquatic-derived carbon in the diets of riparian birds
Source: PLoS One. 2018 Nov 27;13(11):e0207389. doi: 10.1371/journal.pone.0207389 (PMC6258477; doi:10.1371/journal.pone.0207389)

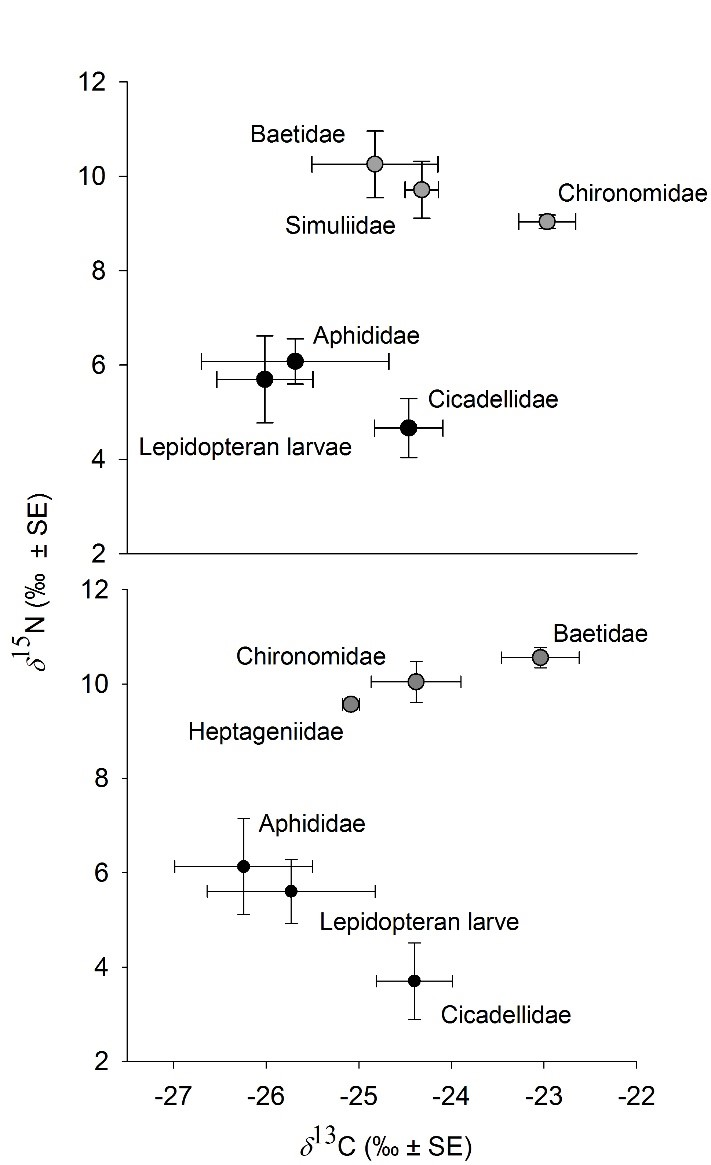

Supplement: S1 Fig — Biplots showing δ13C and δ15N signatures of aquatic (gray) and terrestrial insect taxa (black) used as an index of songbird diet sources in 2015 (top) and 2016 (bottom). Common names of taxa are displayed near the mean. (TIF) [file pone.0207389.s003.tif]
